# Glioblastoma research: US and international networking achievements

**DOI:** 10.18632/oncotarget.21270

**Published:** 2017-09-26

**Authors:** David A. Groneberg, Anna-Maria Addicks, Michael H. Bendels, David Quarcoo, Jenny Jaque, Dörthe Brüggmann

**Affiliations:** ^1^ Division of Epidemiology, Institute of Occupational Medicine, Social Medicine and Environmental Medicine, Goethe-University, Frankfurt, Germany; ^2^ Department of Obstetrics and Gynecology, Keck School of Medicine of USC, Los Angeles, CA, USA

**Keywords:** glioblastoma, network, bibliometry, architecture, structure

## Abstract

Being the most aggressive type of brain tumor, glioblastoma is estimated to be diagnosed in about 12,400 new cases in 2017. The diagnosis is dramatic to patients and relatives and leaves open many unanswered questions for them. One is the big question why there is no cure as in other tumors. This review illustrates the US and global research efforts that have been made over the past century. It demonstrates the great magnitude of energy invested by US clinicians and scientists but undoubtedly, more research is needed and funding by NIH and other sources should be continued on the same level.

## INTRODUCTION

US-American senator John McCain´s diagnosis of glioblastoma left the nation in great sorrow and moved his fellow senators deeply, who prayed for him and his family. With an estimated 12,400 new cases being expected for 2017 by the Central Brain Tumor Registry this is the most common malignant brain tumor in the United States (US) [1]. Like most brain tumors types, the exact cause of glioblastoma is not known [2] leaving patients and their relatives in a desperate situation. A look into the most prestigious scientific journals such as the New England Journal of Medicine [3], Lancet [4, 5] or Journals of the American Medical Association [6] reveals an impressive number of scientific publications that have been published on this condition to date. A large number of basic and clinical studies have also been published in Oncotarget recently [7–19]. However, patients and their families are traumatized by the diagnosis. They have essential questions, which are also commonly shared by lawmakers and donors involved in healthcare policies and research funding: 1) Does a cure exist? 2) If not, why not? 3) Might there be a cure available in the next months? 4) What has the scientific community done to find new treatments? Have they done enough?

On the basis of a bibliometric platform that has been established a decade ago [20], we want to provide answers for the concerned public. This centenary review will share important first insights into the US-American and global achievements in the field of glioblastoma research. Our data reflect quantitative and qualitative aspects, chronological developments as well as collaborative networks and cover the time period from 1900 to 2008.

### Great US research efforts from a global perspective

We used the New Quality and Quantity Indices in Science (NewQIS) platform [20, 21] to assess and visualize research productivity in an objective, reliable and standardized way. The platform combines scientometric methods and “density equalizing mapping projections“ (DEMP) based on algorithms of Gastner and Newman [22] to draw anamorphic maps reflecting analyzed parameters.

A total of 14,411 publications were identified in the Web of Science using the search term “glioblastom^*^”. The first article was identified in 1927; single publications followed in 1933, 1936, 1938 und 1940. Until the late 1960s we found a minor publication activity with less than ten articles published per year. In the next three decades the research output grew moderately; in 1990 authors published 76 publications per year. We noted the first dramatic increase in 1995 with 486 annual publications. Other productivity peaks followed in 1999 (720 annual articles) and 2008 (1500 annual publications).

**Figure 1:**
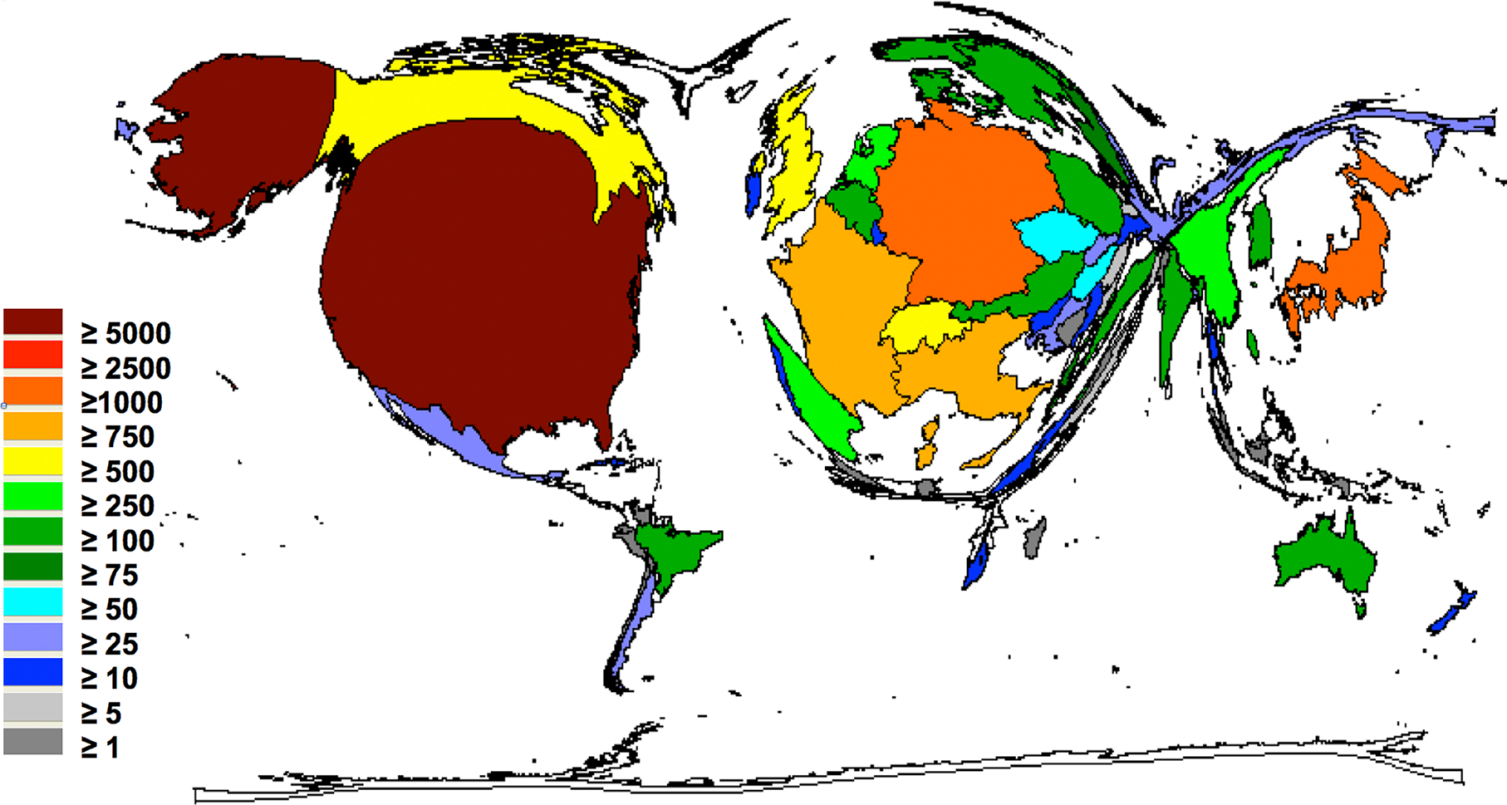
The global glioblastoma research activity Density equalizing mapping of the global glioblastoma research activity assessed by publication output between 1900 and 2008. Colors and territorial sizes indicate numbers of glioblastoma publications per country.

Density equalizing procedures depict the domination of the global glioblastoma research by US scientists having authored a total of 6342 publications. The second most active country was Germany with 1937 glioblastoma-related articles followed by Japan (1422 articles). Research activity outside Northern America and Europe was extremely limited, e.g. authors from China, India and Brasil issued 278, 122 and 120 articles, respectively (Figure 1).

**Figure 2:**
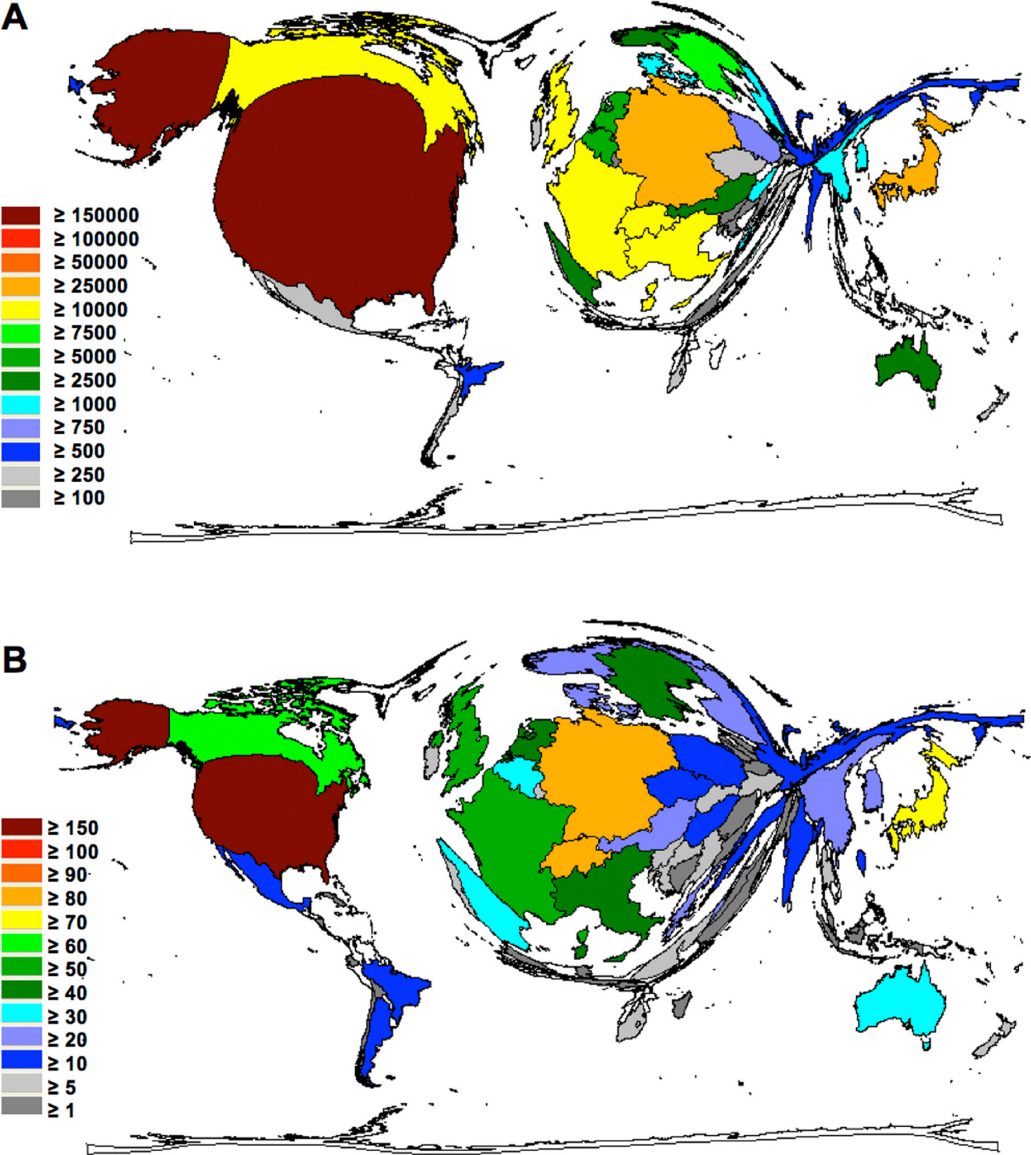
Density equalizing maps of the global glioblastoma research quality (**A**) Colors and territorial sizes indicate total number of citations per country (**B**) Colors and territorial sizes indicate levels of glioblastoma-specific h-indices of countries

How often was this research cited by other publications? This is a surrogate for the quality of research. When the total number of citations was analyzed, the Unites States (US) again occupied the leading position. US-American publications were cited more than 180,000 times, indicating that the scientific community is heavily engaged in discussing the results obtained by US clinical and experimental research. Authors cited German articles 43,728 times, and Japanese articles 25,343 times. Also, articles by authors from Switzerland (21,503), France (14,562), the UK (13,493) Italy (12,194) and Canada (18,884) were well discussed (Figure 2). When the so called h-index was analyzed, the US glioblastoma research received the highest values with a h-index of more than 150, followed by Germany (87) and Switzerland (81). This specific index was named after J. Hirsch, who proposed a novel measure to quantify research output in 2005 [23, 24], and was adapted here to characterize the publication output of countries.

**Figure 3:**
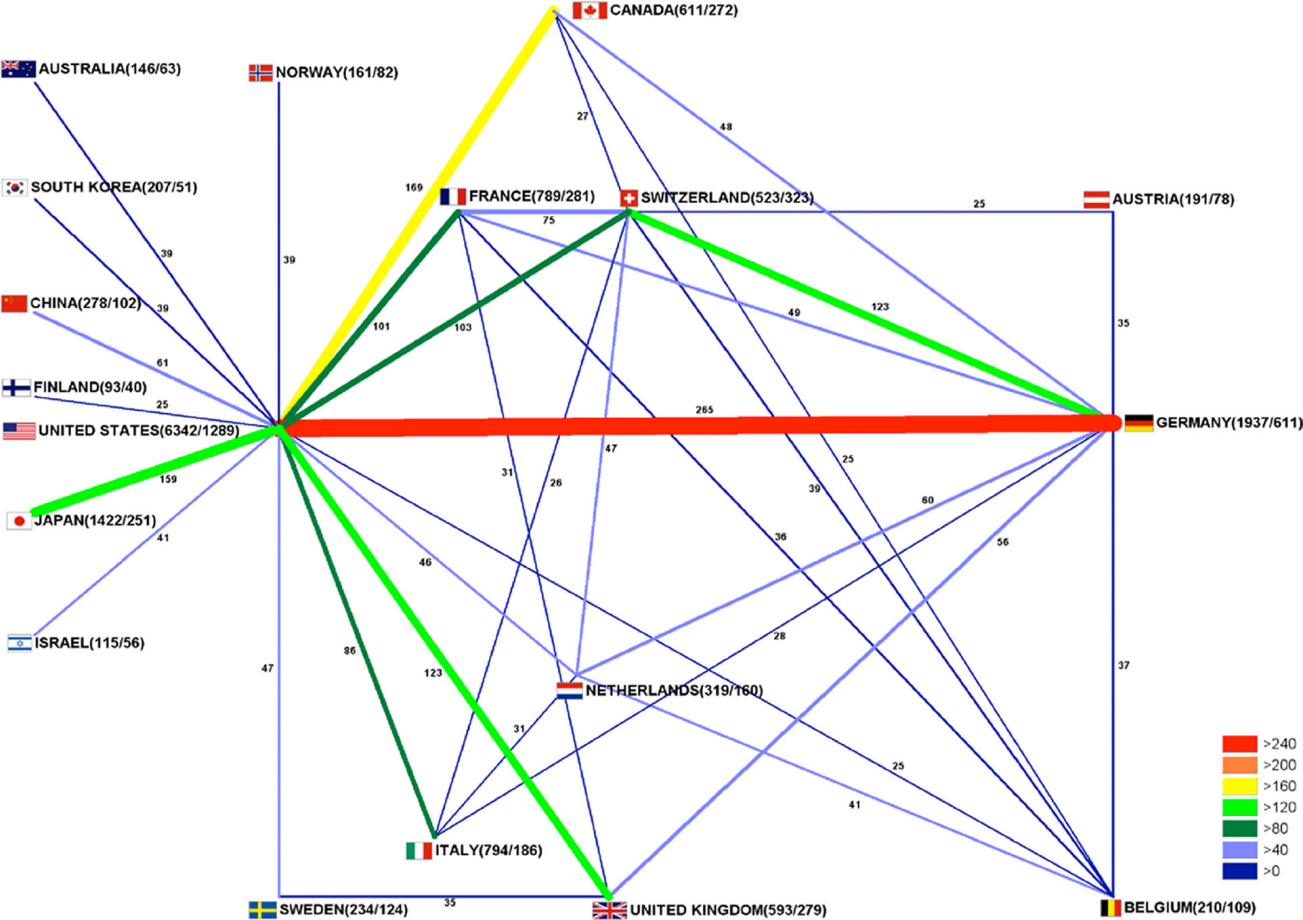
International glioblastoma research collaborations Bar thickness indicates intensity of collaborations. First ciphers in brackets indicate total publication numbers. Second ciphers indicate number of collaborative publications.

Impactful research is commonly produced in a joint effort. Ideally, the best clinicians and researchers come together to share resources and ideas aiming to advance the knowledge in the field. Therefore, insights into the global network of glioblastoma research are important. To analyze the glioblastoma research collaborations from a global perspective, affiliations of all authors were processed as earlier described [25, 26]: In brief, if two authors or more, originating from different countries, contributed to a glioblastoma-specific publication, this collaborative effort was recorded and visualized in a network diagram (Figure 3). Interactions between single pairs of countries were depicted by vectors, which were proportional in shades of grey and width to the number of bilateral collaborations [25, 26]. Clearly, the US constituted the nucleus of global collaborative networks on glioblastoma research. In total, 1,289 out of all 6,342 US-American publications were performed within an international collaboration. Most prominent were collaborations between US-American and German authors (265 publications) followed by joint efforts between the US and Canada (169 publications), US and Japan (159 publications) and US and the United Kingdom (123 publications). In the past, international research was often performed bilaterally between neighboring countries. However, here we found the trend towards research collaborations between countries located far from each other. These sophisticated international networks are transcontinental and not limited by country borders. Collaborations are facilitated by modern communication systems and new computer technologies like the World Wide Web. These tools allow to communicate, exchange ideas and publicize articles in order to advance research in this field for the benefit of the patients and their families.

**Figure 4:**
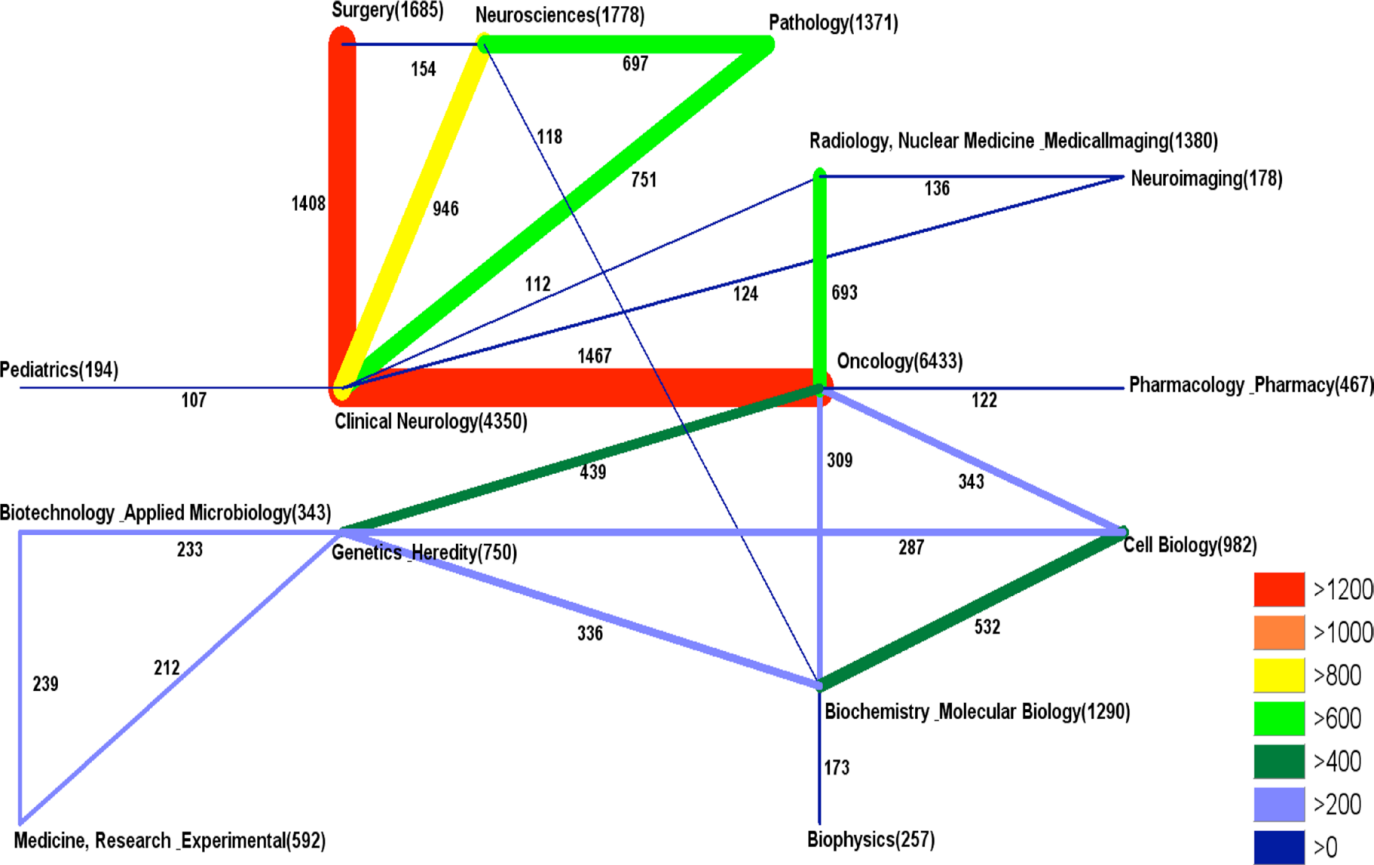
Glioblastoma research subject area analysis

Which are the areas of highest research activity? This question is important for patients and also for donors. The subject areas of clinical neurology and oncology were the most prominent areas. This finding was not surprising. As depicted in Figure 4, there was a considerable overlap between these two fields. This finding indicates numerous interdisciplinary approaches, which also included the important aspects of surgery and diagnostics. Other prominent research fields include the areas of genetics, cell biology, or biochemistry and molecular biology. A high activity in the basic sciences is highly beneficial for the filed since it indicates a continuous interest of scientists to characterize pathogenetic mechanisms and to identify potential new therapeutic targets.

A further important question is: How did the funding landscape change throughout the years. From the present scientometric analysis, it is difficult to answer this question since funding sources of the 14,411 publications are not clearly indicated in every case in the database. Unfortunately, there are no registers that list all relevant project grants. However, single homepages list specific funding. A good example is the National Brain Tumor Society homepage (http://braintumor.org/advance-research/funded-research-and-accomplishments/) that states [27]: “Since 1992, the National Brain Tumor Society has supported brain tumor research by directly funding grants itself, as well was collaborating with other funding organizations on various projects, including: the National Cancer Institute (NCI) Specialized Programs of Research Excellence (SPORE), the American Association for Cancer Research (AACR), the American Association of Neurological Surgeons (AANS), The Bridge Project, and the Brain Tumor Funders’ Collaborative, and the Brain Tumour Foundation of Canada (…) It awarded more than $31 million across 244 grants and grants have gone to organizations in: 30 of the 50 US states, Canada, Israel, The Netherlands. States that received the most funding have been: California, Massachusetts, North Carolina, New York, Pennsylvania, and Texas. The MD Anderson Cancer Center in Houston has received the most individual grants at 22. 68% of the grants went to adult brain tumor research, while 22% went to pediatric brain tumor research. The largest percentage of the grants (59%) went to the study of malignant gliomas (astrocytoma, brain stem glioma; DIPG, glioblastoma multiforme, oligodendroglioma, etc.) – the most dangerous brain tumors – and the rest were spread out among other tumor types” [27]. Ideally, a future study should assess global funding of glioblastoma research systematically. This can provide further rationale why f continuous funding to support glioblastoma research is needed.

## CONCLUSIONS

In summary, this article represents the first centenary assessment of the world-wide glioblastoma research architecture. Using density-equalizing mapping techniques in combination with research quantity and quality indices, a global landscape of glioblastoma research was illustrated. Our data depicts an enormous research activity, which was mainly driven by US scientists and clinicians. Future research in the area should be fostered encompassing molecular biology [28–30], biochemistry [31, 32], morphologic [33–35], and pharmacologic [36, 37] techniques.
